# Effect of axial length and age on the visual outcome of patients with idiopathic epiretinal membrane after pars plana vitrectomy

**DOI:** 10.1038/s41598-019-55544-6

**Published:** 2019-12-13

**Authors:** Sakiko Minami, Hajime Shinoda, Yuta Shigeno, Norihiro Nagai, Toshihide Kurihara, Kazuhiro Watanabe, Hideki Sonobe, Hitoshi Takagi, Kazuo Tsubota, Yoko Ozawa

**Affiliations:** 1 Department of Ophthalmology, Keio University School of Medicine, Tokyo, Japan; 2 Laboratory of Retinal Cell Biology, Department of Ophthalmology, Keio University School of Medicine, Tokyo, Japan; 3 Department of Ophthalmology, Inagi Municipal Hospital, Tokyo, Japan; 4 Department of Ophthalmology, St. Marianna University School of Medicine, Kawasaki, Japan

**Keywords:** Retinal diseases, Medical research

## Abstract

We evaluated predictive factors for visual outcomes in patients with idiopathic epiretinal membrane (iERM) after pars plana vitrectomy (PPV). Clinical records for 114 eyes (114 patients, mean age: 70.6 years) with iERM treated by PPV between March 2012 and March 2018 were retrospectively reviewed. Overall, the mean postoperative best-corrected visual acuity (BCVA) and central retinal thickness measured by optical coherence tomography improved as early as 1 month after surgery, and further improved until 3 months (P < 0.01). Multiple linear regression analyses adjusted for the preoperative BCVA showed that older age (B, 0.010; 95% confidence interval, 0.003 to 0.016; P = 0.003) and a shorter axial length (AL; B, −0.059; 95% confidence interval, −0.099 to −0.019; P = 0.005) predicted worse postoperative BCVA. The Mann-Whitney U test showed that the postoperative BCVA was worse in eyes with AL < 23.6 mm than in eyes with AL ≥ 23.6 mm (P = 0.037), and in patients aged ≥69 years than in patients aged <69 years (P = 0.024). The findings may help in evaluating surgical indications for each patient to obtain satisfactory outcomes, irrespective of the preoperative BCVA.

## Introduction

Recent progress in medical technology has improved the safety of pars plana vitrectomy (PPV), which has led to expansion in its surgical indications. PPV is not only performed for vision-threatening retinal diseases, such as proliferative vitreoretinopathy and proliferative diabetic retinopathy, but also for improving the quality of vision in patients with idiopathic epiretinal membrane (iERM), which has a relative indication of surgery. Thus, in patients with iERM, predictive factors of treatment outcomes would be valuable in choosing the application and/or timing of surgery and achieving satisfying outcomes.

iERM is commonly observed in adults, and its incidence in the primary eye is 1.1% per year. The mean age of iERM diagnosis is 65 years^1^. It has been hypothesized that residual cortical vitreous secondary to a posterior vitreous detachment or partial separation are modified by glial cell proliferation on the surface, forming iERM. While metamorphopsia, blurred vision, monocular diplopia, and micropsia may be noted with any macular pathology due to the tractional force of iERM^2–5^ to the retina, majority of patients with iERM are asymptomatic. Thus, iERM could be occasionally detected using optical coherent tomography (OCT). In contrast, a subtle but clear impairment of visual function can be detected by measuring functional visual acuity and contrast visual acuity of eyes with iERM, even if eyes had best-corrected visual acuity (BCVA) better than 0 in LogMAR (1.0 in decimal in the Landolt C charts, equal to 20/20 in Snellen)^6^.

A previous report showed that better preoperative BCVA may lead to better postoperative BCVA, most likely due to less pathological changes in the retina before surgery^7^. Other reports have shown that disruptions of the integrity of the photoreceptor inner/outer segment layer^8–11^, and photoreceptor damage due to tractional forces of iERM were related to worse visual outcomes after PPV. Therefore, surgery should be performed before photoreceptor changes become evident on OCT images. However, surgeries for subclinical iERM with no clear symptoms may not be accepted, considering the risk-benefit ratio. Fundamental predictive factors related to eye characteristics, other than disease stage, such as the preoperative BCVA and photoreceptor damage detected by OCT findings, would help determine surgical indications in patients with iERM in the recent trend of early treatment than recommended previously.

iERM is formed in the vitreoretinal interface and the tractional force to the retina is most likely influenced by the change in the vitreous^12,13^. Previous observations in eyes with high myopia^14,15^ and posterior staphyloma^16^ show the differences in the vitreous conditions. Vitreous gel may be detached from the outermost layer of the cortex in the eye and observed as vitreous schisis with a longer axial length (AL). Thus, the vitreous condition may have differences according to the size of the eye.

In the present study, we analysed the effect of preoperative clinical data on the early postoperative BCVA in patients who underwent PPV for iERM. In particular, we focused on AL, which does not depend on the stage of the disease and may cause differences in the tractional force of vitreous on the retina.

## Results

**Figure 1:**
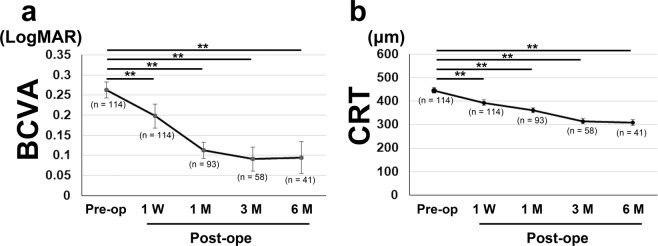
Mean best-corrected visual acuity (BCVA) and mean central retinal thickness (CRT) at each time point. (**a,b**) Generalized mixed model analysis was performed for comparing the preoperative data with the 1-week and 1-, 3-, and 6-month postoperative data. Overall, the mean BCVA (**a**) and CRT (**b**) were significantly improved after surgery. Data are shown in mean ± standard error. **P < 0.01.

Of the 114 patients (114 eyes), 43 (37.7%) were males, and the mean age was 70.6 ± 0.7 years (range, 56–89 years) (Table 1). All patients visited the clinic 1 week after the surgery, and those with a good clinical course were referred back to their original clinics. Thus, the numbers of participants were 114, 93, 58, and 41 at 1 week and 1, 3, and 6 months after the surgery, respectively. The mean BCVA (LogMAR) before surgery, 1 week, and 1, 3, and 6 months after surgery were 0.26 ± 0.020 (range, −0.079 to 1.301; n = 114), 0.20 ± 0.032 (n = 114), 0.11 ± 0.022 (n = 93), 0.09 ± 0.030 (n = 58), and 0.09 ± 0.040 (n = 41), respectively (Fig. 1a). The mean central retinal thickness (CRT) before surgery, 1 week, and 1, 3, and 6 months after surgery were 446.40 ± 12.24 μm (n = 114), 393.81 ± 10.97 μm (n = 114), and 361.00 ± 10.91 μm (n = 93), 315.44 ± 12.01 μm (n = 58), and 309.37 ± 13.51 μm (n = 41), respectively (Fig. 1b). BCVA and CRT were significantly improved relative to the preoperative values at 1 week and 1, 3, and 6 months after surgery (P < 0.01 for all). There were no major complications during and after surgery, and no eyes underwent gas injections.

**Table 1:** Baseline Characteristics.

Mean Age (yrs, range)	70.6 ± 0.7 (56 to 89)
Gender (male, %)	43 (37.7)
Mean BCVA (logMAR, range)	0.26 ± 0.02 (−0.079 to 1.301)
Mean CRT (μm, range)	446.40 ± 12.24 (139 to 738)
Mean IOP (mmHg, range)	13.50 ± 0.30 (7.0 to 21.0)
Mean Axial Length (mm, range)	24.01 ± 0.12 (21.78 to 26.45)
Cystoid changes in inner layer (eyes, [%])	74 (64.9)
Cystoid changes in outer layer (eyes, [%])	33 (28.9)
Cotton Ball Sign at the fovea (eyes, [%])	34 (29.8)
Complications	
Glaucoma (eyes, [%])	9 (7.9)
Hypertension (patients, [%])	13 (11.4)
Diabetes (patients, [%])	9 (7.9)

Data are shown in mean ± SE with range for continuous valuable.

Simple linear regression analyses showed that older age (respective P values at the 1 and 3 months were 0.007 and 0.007), worse preoperative BCVA (P < 0.001 and P < 0.001), shorter AL (P = 0.005 and P = 0.018), and the preoperative presence of cystoid changes in the inner retinal layer (P = 0.031 and P = 0.044) were related to worse BCVA at 1 and 3 months after surgery (Table 2). Greater preoperative CRT was related to worse visual outcome after 1 month (P = 0.036); however, there was no significant association at 3 months (P = 0.059) despite a trend. Older age (P = 0.002), worse preoperative BCVA (P < 0.001), greater preoperative CRT (P = 0.037), and shorter AL (P < 0.001) were also related to worse visual outcome, 1 week after the surgery (data not shown).

**Table 2:** Correlation Between Baseline Parameters and postoperative best-corrected visual acuity at Month 1 and 3.

Variables	Month 1		Month 3	
	B (95% CI)	P Value	B (95% CI)	P Value
Age	0.008 (0.002 to 0.014)	0.007**	0.011 (0.003 to 0.018)	0.007**
Gender	−0.003 (−0.103 to 0.097)	0.958	0.032 (−0.090 to 0.155)	0.602
Preoperative BCVA	0.578 (0.426 to 0.730)	<0.001**	0.426 (0.236 to 0.615)	<0.001**
Preoperative CRT	0.000 (0.000 to 0.001)	0.036*	0.000 (0.000 to 0.001)	0.059
Preoperative IOP	0.010 (−0.007 to 0.028)	0.242	0.015 (−0.006 to 0.037)	0.159
Axial Length	−0.053 (−0.090 to −0.017)	0.005**	−0.058 (−0.106 to −0.011)	0.018*
Cystoid changes in inner layer	0.113 (0.011 to 0.215)	0.031*	0.136 (0.004 to 0.276)	0.044*
Cystoid changes in outer layer	−0.021 (−0.127 to 0.084)	0.688	0.004 (−0.132 to 0.141)	0.948
Cotton Ball Sign	−0.030 (−0.132 to 0.072)	0.561	−0.055 (−0.177 to 0.067)	0.370

Simple linear regression analyses. BCVA, best-corrected visual acuity; CRT, central retinal thickness; IOP, intraocular pressure. *P < 0.05, **P < 0.01.

Multiple linear regression analyses adjusted for the preoperative BCVA were performed by referring to the results of the simple linear regression analyses (Table 3). The selected factors that were statistically significant in univariate analyses are shown in Table 2. After adjusting for the preoperative BCVA (Table 3, Supplementary Fig. 1), we found that worse visual outcomes at 1 month and 3 months were associated with older age (respective P values at 1 month and 3 months were 0.005 and 0.003), and shorter AL (P = 0.002 and P = 0.005). The other factors did not show significant relationships. The same results were also observed at 1 week (P = 0.001 for both, data not shown).

**Table 3:** Predictors of visual outcome at Month 1 and 3 after pars plana vitrectomy in idiopathic epiretinal membrane.

Variables	Month 1			Month 3		
	B (95% CI)	β	P Value	B (95% CI)	β	P Value
Age	0.007 (0.002 to 0.012)	0.228	0.005**	0.010 (0.003 to 0.016)	0.328	0.003**
Axial Length	−0.046 (−0.075 to −0.017)	−0.248	0.002**	−0.059 (−0.099 to −0.019)	−0.315	0.005**

Multiple linear regression analyses adjusted for preoperative BCVA. The selected factors were age, axial length, preoperative CRT and cystoid changes in inner layer. The R square values for Months 1 and 3 were 0.44 and 0.37, respectively. **P < 0.01.

**Figure 2:**
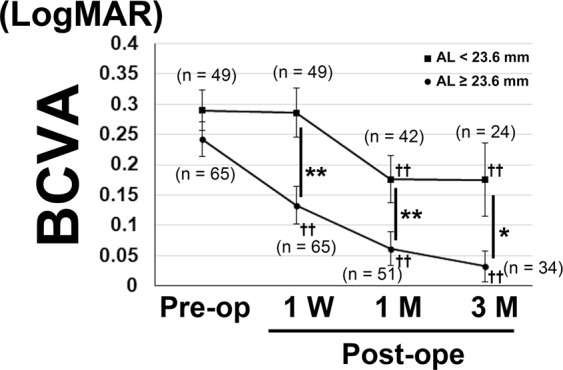
Mean best-corrected visual acuity (BCVA) in the patients with axial length (AL) < and ≥23.6 mm. Mann-Whitney U test was performed for comparing mean BCVA at each time point between the groups. In patients with AL <23.6 mm, the mean BCVAs were significantly worse at 1 week and 1- and 3-months postoperatively. Generalized mixed model analysis was performed for comparing the preoperative data with the 1-week and 1- and 3-month postoperative data. BCVA improved at 1 week after surgery in patients with AL ≥23.6 mm, while it was better than the preoperative value at 1 and 3 months after surgery in both groups. Data are shown as mean ± standard error. *P < 0.05, **P < 0.01 for comparisons between the groups. ^††^P < 0.01 for comparisons between preoperative and postoperative values at each time point.

We subsequently divided the patients by their AL to find the cut-off value, which reflects the visual outcome (Fig. 2). In patients with AL < 23.6 mm, the postoperative mean BCVA was significantly worse than that in patients with AL ≥ 23.6 mm at 1 week and 1 and 3 months after surgery (P < 0.001, P = 0.001, P = 0.037 respectively), whereas the preoperative BCVA was not different between the two groups (P = 0.203). The mean postoperative BCVA in patients with AL ≥ 23.6 mm was better than the preoperative value at all time points. Other characteristics of patients with AL < 23.6 mm included an older age at the time of surgery and female predominance. However, there were no differences in the presence of preoperative OCT findings such as the cotton ball sign between patients with AL < 23.6 mm and those with AL ≥ 23.6 mm (Table 4, Supplementary Fig. 1). Gender was reanalysed using multiple linear regression analyses adjusted for the preoperative BCVA; however, it showed no association with the postoperative BCVA at 1 (P = 0.278) and 3 (P = 0.138) months (data not shown).

**Table 4:** Comparison of Parameters in Patients with Axial Length <23.6 mm and ≥23.6 mm.

Variables	AL < 23.6 mm	AL ≥ 23.6 mm	P value
Baseline			
Age	74.31 ± 0.99	67.74 ± 0.87	<0.001**
Gender (male, [%])	8 (16.3)	35 (53.8)	<0.001**
Preoperative BCVA	0.290 ± 0.03	0.242 ± 0.03	0.273
Preoperative CRT	444.55 ± 20.42	447.80 ± 15.09	0.896
Cystoid changes in inner layer (eyes, [%])	35 (71.4)	39 (60.0)	0.206
Cystoid changes in outer layer (eyes, [%])	15 (30.6)	18 (27.7)	0.734
Cotton Ball Sign at the fovea (eyes, [%])	12 (24.5)	22 (33.8)	0.280
Intra- and Post-operative Complications			
Retinal breaks	2 (4.1)	7 (10.8)	0.296
Retinal hemorrhage	3 (6.1)	1 (1.5)	0.313
Vitreous hemorrhage	1 (2.0)	0 (0)	0.430
Postoperative refractive error	−0.73 ± 0.09	−1.49 ± 0.21	0.003**

Data are shown in mean ± SE. Student’s t-test, chi-square test, and Fisher’s exact test. Data of posterior vitreous detachment was obtained from surgical record at the beginning of the surgery. BCVA, best-corrected visual acuity; CRT, central retinal thickness. **P < 0.01.

With regard to age, patients aged ≥69 years exhibited worse postoperative BCVA than did those aged <69 years at 1 week (P = 0.003), and 1 (P = 0.010) and 3 (P = 0.024) months (number of patients with age ≥69 years and <69 years: 50 and 43 at 1 month and 30 and 28 at 3 months; Supplementary Fig. 2).

We also divided the patients into 4 groups on the basis of AL (<23.6 mm or ≥23.6 mm) and age (<69 or ≥69), and found significant differences among the four groups (Table 5). Although the preoperative BCVA showed no significant differences, the postoperative BCVA was worse in patients with AL < 23.6 mm and age ≥69 years than in those with AL ≥23.6 mm and age <69 years at 1 (P = 0.016) and 3 (P = 0.031) months.

**Table 5:** Mean best-corrected visual acuity for patients stratified by age and axial length.

Group	1	2	3	4	P value
Axial Length (mm)	<23.6	<23.6	≥23.6	≥23.6	
Age (yrs)	<69	≥69	<69	≥69	
Preoperative BCVA	0.341 ± 0.10	0.276 ± 0.03	0.206 ± 0.03	0.286 ± 0.05	0.288
Eyes	11	38	36	29	
Postoperative BCVA					
Month 1	0.196 ± 0.10	0.170 ± 0.04	0.001 ± 0.02	0.170 ± 0.06	0.006**
Eyes	10	32	33	18	(0.016*)
Month 3	0.134 ± 0.15	0.192 ± 0.06	−0.016 ± 0.02	0.110 ± 0.05	0.038*
Eyes	7	17	21	13	(0.031*)

Data are shown as mean ± SE. P values for differences among the four groups were derived by one-way analysis of variance. Post hoc Bonferroni correction analyses were applied for differences between groups 2 and 3 (P values in parentheses). *P < 0.05, **P < 0.01.

**Figure 3:**
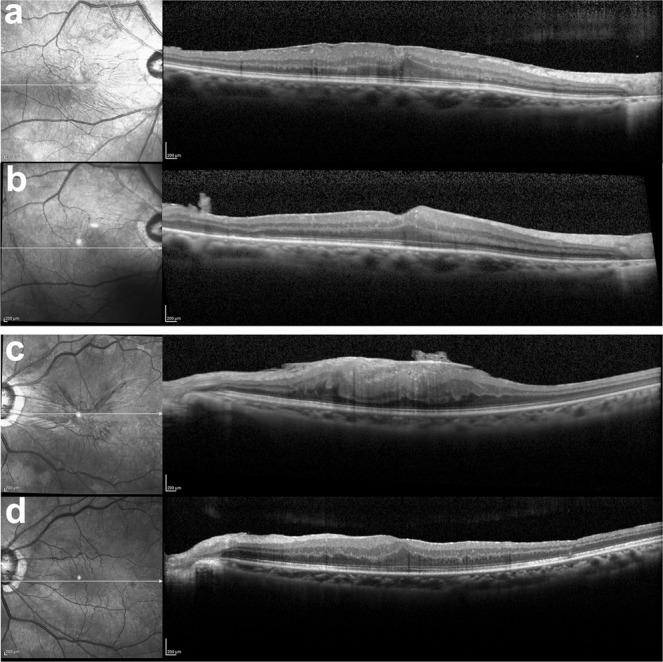
Representative preoperative optical coherent tomography (OCT) images of the eyes with idiopathic epiretinal membrane and an axial length (AL) < and ≥23.6 mm. (**a,b**) A 75-year-old woman exhibited an AL of 22.6 mm and worse postoperative BCVA relative to the average value (preoperative: 0.155, postoperative: 0.699 at 1 month and 0.523 at 3 months). (**c,d**) A 63-year-old woman exhibited an AL of 25.6 mm and good postoperative BCVA (preoperative: 0.097, postoperative: −0.079 at 1 and 3 months). Preoperative (**a,c**) and postoperative (**b,d**) OCT images at 1 month.

Representative data for patients with shorter and longer ALs are shown in Fig. 3(a–d). A 75-year-old woman exhibited an AL of 22.6 mm and worse postoperative BCVA relative to the average value (preoperative: 0.155, postoperative: 0.699 at 1 month and 0.523 at 3 months; Fig. 3a,b). In contrast, a 63-year-old woman exhibited an AL of 25.6 mm and good postoperative BCVA (preoperative: 0.097, postoperative: −0.079 at 1 and 3 months; Fig. 3c,d).

## Discussion

In the present study, we found that patients with iERM exhibited an overall improvement in BCVA after PPV; however, the mean outcome value was worse for patients with a shorter AL and an older age after adjustment for the preoperative BCVA. Eyes with AL ≥ 23.6 mm and patients aged <69 years exhibited a significantly better postoperative BCVA than did eyes with AL < 23.6 mm and patients aged ≥69 years, respectively.

Simple linear regression analysis showed that better preoperative BCVA was related to better postoperative BCVA, which is consistent with the findings of previous reports^7,9,17–24^. Similarly, better pretreatment BCVA leading to better posttreatment BCVA is often reported in patients with retinal diseases such as diabetic macular oedema^25,26^. This is most likely because better pretreatment BCVA reflects lower levels of retinal neural damage that may be irreversible after treatment. Although the improvement in BCVA may be less in the eyes that had better preoperative BCVA due to the ceiling effect^7,9,20,27^, to obtain better visual outcomes, patients with iERM may benefit more if they undergo surgery before substantial BCVA loss. However, early intervention may not always be successful and lead to overtreatment, considering the risk of surgical complications. However, such complications have become rare with the recent advances in technology.

After adjusting for the preoperative BCVA, older age and a shorter AL affected the visual outcomes. The worse recovery of the retinal condition in older patients may be related to the fundamental vulnerability of the retinal neurons. Both photoreceptor cells^28^ and ganglion cell nerve fibres^21^ are reduced with age under physiological conditions. The tractional force of the iERM before surgery and/or the force at the time of removing iERM during surgery, may easily damage the retinal neurons in older patients. In fact, there is a report that eyes of older patients had a trend of exhibiting postoperative intraretinal cystoid changes, which could reflect neurodegeneration^29–35^ more frequently^36^. Patients with iERM may benefit by undergoing surgery at a relatively younger age. However, older patients also visit the clinics and require surgery. For older patients, when obtaining informed consent, it should be made clear to the patients to not have very high expectations; nonetheless, the patients may still undergo the surgery.

Interestingly, the eyes with a shorter AL had worse postoperative visual outcomes compared with those with a longer AL in iERM. This was significant 1 week after surgery (data not shown), and clearer at 1 and 3 months after surgery, adjusting for the preoperative BCVA. For patients with iERM, if the patient has a shorter AL, clinicians should be more prudent in recommending the surgery.

**Figure 4:**
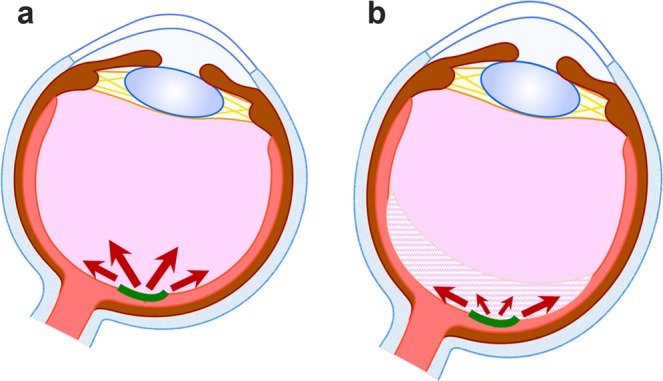
A hypothetical mechanism for the differences related to the tractional force of the retina according to the axial length (AL). (**a**) Eyes with a shorter AL may have undetached or incompletely detached posterior vitreous, which directly mediates vitreous tractional force to the macula chronically (arrows). Under this condition, idiopathic epiretinal membrane (iERM) modification by glial cells and cytokines may occur easily; this further strengthens the adhesion of iERM to the retina and increases the tractional force. In addition, the surgical procedure to intentionally detach the vitreous and/or iERM may mediate greater tractional force to the retina acutely, which induces more damage to the macula. (**b**) In contrast, eyes with relatively longer AL may already have posterior vitreous detachment or vitreous schisis (shaded area), which causes less traction (arrows) and less damage to the macula. iERM is shown in green.

The mechanism of a shorter AL causing worse visual outcomes may be related to the tractional forces at the vitreoretinal interface caused by iERM. A previous study has shown that posterior vitreous detachment (PVD) is less frequently seen in eyes with a shorter AL in diabetic retinopathy patients^37^. Eyes with a shorter AL may have no PVD or macula-sparing PVD, which directly mediates vitreous tractional forces to the macula chronically (Fig. 4a). Moreover, iERM is gradually modified by the migration of Müller glial cells and cytokines such as transforming growth factor-beta from the retina, as reported previously^38^. The cell migration becomes easier when the tractional force is greater, and cytokines are reported to be concentrated in eyes with a shorter AL^39^, most probably because vitreous liquefaction has not progressed much; this slows the turnover of soluble factors^40^. Thus, iERM may have undergone severe modifications in eyes with a shorter AL, and these iERMs may have exhibited stronger adhesion to the retina with greater tractional force. In addition, under these conditions, the surgical procedure for intentionally detaching the vitreous and/or iERM may have acutely exerted a greater tractional force on the retina. These chronic and/or acute tractional forces may damage the neural retina in the macular region substantially by the end of the surgery. This could occur easily in the eyes with a shorter AL. In fact, the sticky adhesion of iERM to the retina or unintended en bloc resection of iERM and the internal limiting membrane (ILM) because of strong adhesion was often observed in the surgical records of patients with a shorter AL. We found these intraoperative findings in the surgical records of 53% eyes with a shorter AL and 20% eyes with a longer AL (as far as we could find the comments by the surgeons). In contrast, eyes with a relatively longer AL may easily develop PVD or vitreous schisis with vitreous liquefaction, which causes lesser traction on the retina and/or lesser modification of the iERM, thus resulting in lesser damage to the macula (Fig. 4b).

The shorter AL group in our study involved older patients; this could be a confounding factor for a worse visual outcome. However, this also indicated that surgery for iERM may be required after a certain age in patients with a shorter AL, probably because of differences in the vitreous condition. Eyes with a shorter AL may take longer to develop iERM related to later PVD occurrence. Given that iERM is of two different types based on the extracellular matrix^41^, differences in the developmental course may result in differences in the components and characteristics of iERM.

The findings related to the outer retina and photoreceptor cells, such as the cystoid changes in the outer retinal layer and cotton ball sign^42^ were not included in the predictive factors of visual outcomes in the current study, although were analysed. This would be because the surgical application for iERM in the current study was early and most eyes had not yet developed these findings by the time of surgery. Therefore, AL, which is not related to the disease stage, could be a valuable factor in determining the surgery considering the recent trend of giving more importance to a satisfaction of the patients. Eyes with a shorter AL may have retinal damages and/or may have been damaged at the time of surgery, making them less likely to have better outcomes than expected. Given that good outcomes cannot be anticipated with the current surgical applications, whether the eyes with a shorter AL should be treated earlier (i.e. before having retinal damage) or treated later (i.e. after BCVA impairment has progressed and intervention has become unavoidable) would be the topic for future discussion.

The limitations of the current study were the small sample size, retrospective study design, and relatively short follow-up duration. However, patients with iERM may have rapid recovery of BCVA under the recent early indication of the surgery. In fact, the mean BCVA reached a plateau at 3 months after surgery, with no change between 3 and 6 months (paired t-test, P = 0.394; data not shown). Also, patients who showed a good clinical course were referred back to their original clinics soon after surgery; thus, the number of participants decreased with time. This may have resulted in selection bias. Furthermore, the presence or absence of PVD was not determined because of the retrospective design. A recent report showed PVD by focusing on the vitreous at the time of scanning using a wide-angle OCT system^43^; thus, a future prospective study is warranted. Although we found surgical records regarding iERM adhesion in many of the patients, they were not from all patients. Although ILM was peeled as far as possible, it may have remained in some patients. Cataract surgery was performed at the same time as PPV as is usual in Japan, although cataract was mild and of the same grade in all patients and most likely caused no differences in the outcomes of the current study.

Early treatment for iERM may be accepted universally considering the significant progress of surgical technology and the better visual outcomes in patients with better preoperative BCVA^7^, as previously reported. However, patients with a shorter AL may not necessarily show the expected postoperative BCVA and may have a limited outcome, most likely because patients with iERM may be susceptible to iERM-related retinal damage. The current study may help in evaluating surgical indications in each patient to obtain better and satisfactory outcomes irrespective of the pathological stage of iERM reflected in the preoperative BCVA, although further long-term studies with an increased number of patients are warranted.

## Methods

### Patients

This was a retrospective study performed at the Vitreo-Retina Surgical Division Clinic of the Department of Ophthalmology at Keio University Hospital. Informed consent was obtained from all subjects after providing them with an explanation of the procedures to be used in the present study. The procedures adhered to the tenets of the Declaration of Helsinki, and approval to perform this study was obtained from the Keio University School of Medicine Ethics Committee (Approval number: 20100003).

### Inclusion and exclusion criteria

One hundred fourteen eyes of 114 patients who underwent the surgery between March 2012 and March 2018 due to subjective visual disturbances resulting from iERM were studied. The eyes with (1) media opacities, which result in the acquisition of poor OCT images; (2) a secondary ERM caused by conditions such as diabetic retinopathy, retinal vein occlusion, retinal tear and detachment, uveitis, and trauma; (3) a severe cataract of more than grade 3 nuclear sclerosis or cortical opacity; and (4) a high myopia (AL ≥ 26.5 mm) were excluded. High myopia was excluded to avoid the inclusion of pathological myopia.

### Eye examinations

All patients underwent complete ophthalmologic examinations, including measurement of BCVA using a Landolt C chart, biomicroscopy of the fundus, fundus photography, and OCT (see below), pre- and post-operatively. An IOL Master (Carl Zeiss Meditec, CA, USA) was used for preoperative AL measurement.

### OCT

The OCT images were obtained with spectral domain-OCT (Heidelberg Spectralis OCT, Dossenheim, Germany). After dilatation of the pupils, the patients were asked to fixate on a target, and both horizontal and vertical images were recorded at baseline (before the surgery), as well as 1 week and 1, 3, and 6 months after the surgery. The CRT was defined as the distance between the inner retinal surface and the inner border of the retinal pigment epithelium and was measured with the built-in scale in the OCT system. Cystoid changes were defined in the vertical and horizontal OCT images within 1500 μm from the fovea. The inner layer of the retina represents the layers from the internal limiting membrane to the inner nuclear layer, and the outer layer represents the outer plexiform layer to the inner border of the retinal pigment epithelium. The cotton ball sign^42,44^ was defined in the OCT images according to previous reports. The findings were defined by two retina specialists (SM and YO).

### Surgery

In all patients, phacoemulsification with intraocular lens implantation was performed for mild cataract, followed by PPV for iERM with 25-gauge 3-port vitrectomy using forceps by experienced surgeons (HS and YO). Intentional ILM peeling was performed in occasional cases where en bloc resection of ERM and ILM was not performed.

### Statistical analyses

Data are expressed as mean ± SE. Generalized mixed model analyses, Mann-Whitney U test, simple linear regression analyses, multiple linear regression analyses adjusted for the preoperative BCVA using the forced input method, Student’s t-test, chi-square test, Fisher’s exact test, and Pearson correlation coefficient were performed using SPSS software (version 25.0, SPSS Japan, Tokyo, Japan). A P value of < 0.05 was considered statistically significant.

### Approval, accordance and informed consent statements

The study adhered to the tenets of the Declaration of Helsinki, and approval to perform this study was obtained from the Keio University School of Medicine Ethics Committee (Approval number: 20100003). Informed consent was obtained from all the participants.

## Supplementary information

Dataset 1

## Data Availability

The protocol and the datasets generated during and/or analysed during the current study are available from the corresponding author upon reasonable request.
