# Supplementary material for: Effect of axial length and age on the visual outcome of patients with idiopathic epiretinal membrane after pars plana vitrectomy
Source: Sci Rep. 2019 Dec 13;9:19056. doi: 10.1038/s41598-019-55544-6 (PMC6911110; doi:10.1038/s41598-019-55544-6)
Supplement: Supplementary file 1 — Dataset 1 [file 41598_2019_55544_MOESM1_ESM.pdf]

Effect of axial length and age on the visual outcome of patients with idiopathic epiretinal membrane after pars plana vitrectomy

Sakiko Minami[1a,2,3], Hajime Shinoda[1a], Yuta Shigeno[1a], Norihiro Nagai[1ab], Toshihide Kurihara[1a], Kazuhiro Watanabe[1a], Hideki Sonobe[1a], Hitoshi Takagi[3], Kazuo Tsubota[1a], Yoko Ozawa*[1ab]

1a Department of Ophthalmology, Keio University, School of Medicine; 1b Laboratory of Retinal Cell, Biology Department of Ophthalmology, Keio University, School of Medicine; 2 Inagi Municipal Hospital; 3 Department of Ophthalmology, St. Marianna University School of Medicine.

***Corresponding author:**
Yoko Ozawa, M.D., Ph.D.
Laboratory of Retinal Cell Biology
Department of Ophthalmology
Keio University School of Medicine
35 Shinanomachi, Shinjuku-ku, Tokyo 160-8582, Japan
Phone: +81-3-5363-3869, Fax: +81-3-5363-3869
E-mail: ozawa@a5.keio.jp

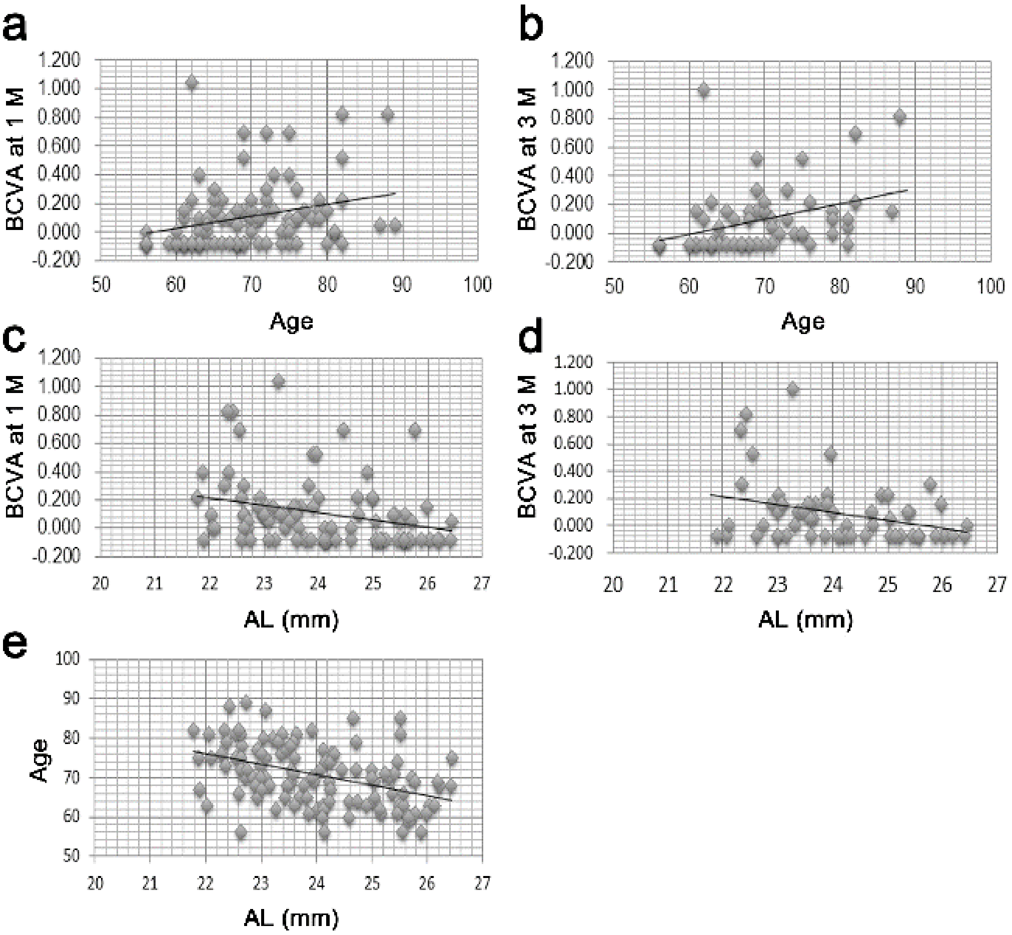

Supplementary Figure 1

Supplementary Figure 1

Scatter plots between best-corrected visual acuity (BCVA), age and axial length (AL)

Pearson correlation coefficient. There were correlations between age and BCVA at 1 month (a, r=0.276, P=0.007), age and BCVA at 3 months (b, r=0.353, P=0.007), AL and BCVA at 1 month (c, r=-0.289, P=0.005), AL and BCVA at 3 months (d, r=-0.311, P=0.018), and age and AL (e, r=-0.428, P<0.001). BCVA, best-corrected visual acuity; AL, axial length.

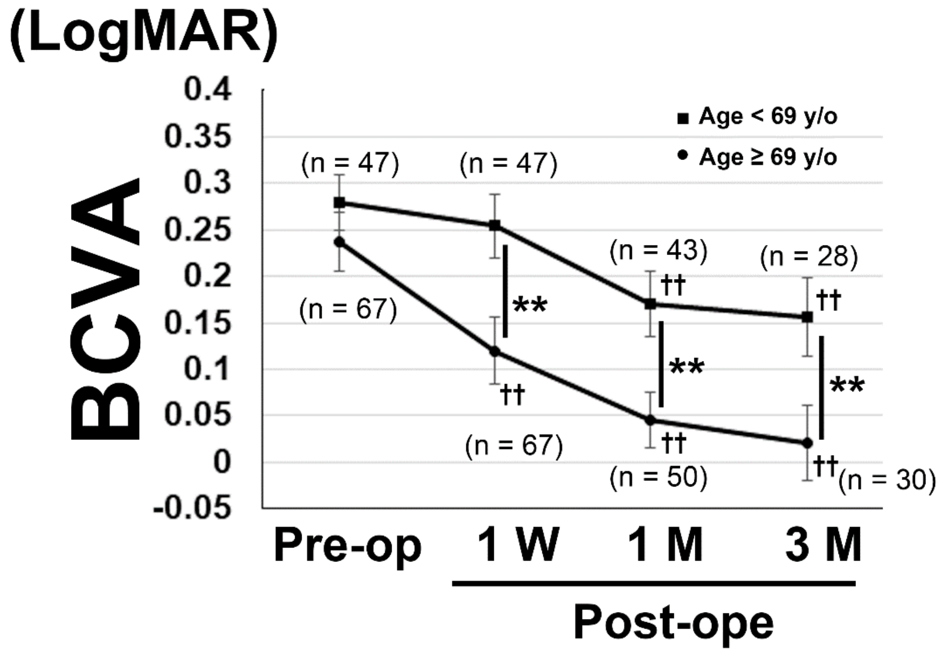

Supplementary Figure 2

Supplementary Figure 2.

Mean best-corrected visual acuity (BCVA) in the patients with age < and ≥ 69 years old

Mann-Whitney U test was performed for comparing mean BCVA at each time point between the groups. In patients with age ≥ 69, the mean BCVAs were significantly worse at 1 week and 1- and 3-months postoperatively. Generalized mixed model analysis was performed for comparing the preoperative data with the 1-week and 1- and 3-month postoperative data. BCVA improved at 1 week after surgery in patients aged < 69 years old, while it was better than the preoperative value at 1 and 3 months after surgery in both groups. Data are shown as mean ± standard error. **P < 0.01 for comparisons between the groups. ††P < 0.01 for comparisons between preoperative and postoperative values at each time point.
